# A structured protocol for Preoperative Progressive Pneumoperitoneum (PPP) in Complex Abdominal Wall Reconstruction (CAWR): our York protocol

**DOI:** 10.1007/s10029-025-03401-z

**Published:** 2025-06-17

**Authors:** Asim A. Abbas, Anisah Ahmad, Alastair McCleary, Marcus Nicholls, Praminthra Chitsabesan, Srinivas Chintapatla

**Affiliations:** 1 Department of General Surgery, York Abdominal Wall Unit, York and Scarborough Teaching Hospitals NHSFT, 4th Floor, Administrative Block, Wigginton Road, Clifton, York, YO31 8HE UK; 2 York Hospital Pharmacy Department, York and Scarborough Teaching Hospitals NHSFT, York, UK; 3 Department of Vascular Surgery, York and Scarborough Teaching Hospitals NHSFT, York, UK; 4 Department of Interventional Radiology, York and Scarborough Teaching Hospitals NHSFT, York, UK

**Keywords:** Progressive pneumoperitoneum, Complex abdominal wall reconstruction, Loss of domain, Ventral hernia, Inferior vena cava filter, Venous thromboembolism (VTE) prophylaxis

## Abstract

**Background:**

Complex Abdominal Wall Reconstruction (CAWR) in patients with significant loss of domain poses substantial surgical and physiological challenges [1], [2]. Preoperative Progressive Pneumoperitoneum (PPP), involving incremental insufflation of gas into the abdominal cavity, enhances the likelihood of tension-free fascial closure [3]. However, there remains considerable variability and incompleteness in existing PPP protocols, especially concerning venous thromboembolism (VTE) prophylaxis, inferior vena cava (IVC) filter placement, respiratory prehabilitation, and multidisciplinary coordination.

**Methods:**

A structured literature review was conducted (MEDLINE® and Embase®, final search April 2025), yielding no comprehensive PPP protocols for CAWR. In response, we convened a multidisciplinary team at a tertiary UK referral centre—including specialists in general surgery, plastic and reconstructive surgery, vascular surgery, interventional radiology, and pharmacy—to develop an integrated, replicable protocol. Consensus development methods (CDMs) informed the iterative refinement process, incorporating clinical experience and best available evidence.

**Results:**

Our seven-week perioperative protocol systematically integrates key preoperative interventions: bilateral abdominal wall botulinum toxin injections, respiratory prehabilitation, abdominal binder use, VTE prophylaxis with low molecular weight heparin (LMWH), planned IVC filter insertion, peritoneal dialysis catheter placement, and scheduled PPP insufflation sessions. Final surgical planning is guided by crosssectional imaging obtained one week preoperatively. Postoperatively, a structured IVC filter removal strategy, including cavogram assessment, is implemented to manage thromboembolic risk.

**Conclusion:**

We present a comprehensive PPP protocol designed to optimise outcomes in CAWR. This structured, multidisciplinary approach represents an important step toward standardising care in complex abdominal wall reconstruction.

## Introduction

Complex Abdominal Wall Reconstruction (CAWR) presents a significant surgical challenge, particularly in patients with large ventral hernias associated with substantial loss of domain. Loss of domain has been defined as “A ventral hernia large enough such that simple reduction in its contents and primary fascial closure cannot be achieved without additional reconstructive techniques or cannot be achieved without significant risk of complications due to the raised intra-abdominal pressure.” [1]. This consensus definition shifts the focus to the clinical complexity rather than just anatomical measurements in loss of domain. In such cases, achieving primary fascial closure can be technically demanding and is associated with elevated risks of postoperative morbidity, including abdominal compartment syndrome, respiratory compromise, and hernia recurrence.

Preoperative Progressive Pneumoperitoneum (PPP), first described by Goñi Moreno in the 1940 s [2], has re-emerged in recent decades as a valuable adjunct in the preoperative optimisation of patients undergoing CAWR. By gradually insufflating gas into the peritoneal cavity over several sessions, PPP facilitates progressive expansion of the abdominal cavity, thereby improving the domain-to-content ratio and increasing the likelihood of tension-free fascial closure [2–4]. Despite its documented benefits, widespread adoption of PPP has been limited by the absence of a standardised and comprehensive protocol that integrates this technique into the broader perioperative framework of CAWR [5]. Complications of PPP include shoulder-tip pain, subcutaneous emphysema, dyspnea, deep venous thrombosis, pulmonary thromboembolism, acute renal failure, respiratory failure, port infection, and death [6].

Current descriptions of PPP methods used in studies show significant variability and are often poorly described, with little consensus on the timing, volume of insufflation, monitoring parameters, or integration with essential preoperative and perioperative considerations [7, 8]. Importantly, aspects such as venous thromboembolism (VTE) prophylaxis, inferior vena cava (IVC) filter insertion, respiratory prehabilitation, and coordination with surgical planning are frequently omitted or inconsistently reported in the literature.

In this manuscript we describe a practical, comprehensive, time-structured protocol for the implementation of PPP in the context of CAWR. Developed and refined at our institution through multidisciplinary collaboration, this protocol encompasses not only the technical aspects of PPP but also addresses adjunctive measures critical to optimising patient outcomes. These include botulinum toxin injections to the abdominal wall, respiratory muscle training, IVC filter insertion, tailored VTE prophylaxis, and coordinated imaging and operative planning. To our knowledge, this is the first published protocol to systematically integrate all these elements into a unified perioperative strategy. By presenting our protocol in detail, we aim to provide a practical, reproducible framework for centres considering the implementation of PPP in complex hernia care.

## Methods/Protocol development

To assess the current landscape of published protocols for PPP in the context of CAWR, we conducted a structured literature search using MEDLINE® and Embase®, with the final search performed on 8 April 2025. Search terms included combinations of “progressive pneumoperitoneum,”, “progressive preoperative pneumoperitoneum”, “complex abdominal wall reconstruction,” “hernia,” “protocol,” and “preoperative optimisation.” This search yielded 15 unique records.

Despite the increasing interest in PPP as an adjunct to complex hernia repair, our search did not yield any comprehensive, time-based protocols that systematically incorporate all aspects of perioperative care. While several case series and technical notes described the utility of PPP in isolated settings, and we identified a number of systematic reviews commenting on indication and technique, we identified no publications that provided a fully integrated, multidisciplinary framework encompassing VTE prophylaxis, IVC filter management, respiratory prehabilitation, operative planning, and postoperative management.

This absence of a unified protocol highlighted a significant gap in the literature and served as the rationale for developing a formalised and replicable protocol to guide PPP implementation in CAWR.

In response to this gap, we initiated a structured protocol development process at a tertiary hernia referral centre in the United Kingdom, where multidisciplinary input and access to advanced radiological and surgical support are routinely available. Patients referred to the service often present with large or recurrent hernias, prior failed repairs, and significant comorbidities, necessitating bespoke preoperative strategies one of which is PPP. The development of the protocol was informed by clinical experience, existing literature on PPP and CAWR, and iterative feedback from relevant specialties. A multidisciplinary working group was convened and included representatives from: General Surgery (with expertise in abdominal wall reconstruction); Plastic and Reconstructive Surgery (with expertise in abdominal wall reconstruction); Vascular Surgery (to advise on IVC filter indications and retrieval); Interventional Radiology (responsible for peritoneal catheter placement if not done surgically and IVC filter insertion/removal); and Pharmacy (Lead Pharmacist for Anticoagulation, to advise on pharmacological VTE prophylaxis, including weight-based dosing and renal function considerations).

Patients were considered eligible for the protocol if they had large complex ventral hernias with significant loss of domain, as assessed clinically and via cross-sectional imaging, with loss of domain calculated using the Tanaka method at our institution [9], and were being planned for elective abdominal wall reconstruction [1, 10]. Patients with contraindications to pneumoperitoneum (e.g. untreated intra-abdominal sepsis, uncorrected coagulopathy), unsuitable anatomy for catheter placement, or severe cardiopulmonary compromise based on physiological and anaesthetic assessment were excluded.

The team met at regular intervals over a six-month period to synthesise the protocol. This development process followed principles of consensus development methods (CDMs), as outlined by Arakawa and Bader, which advocate for systematic and transparent group decision-making in health system frameworks where evidence is limited or evolving. While we did not adopt a formal Delphi or Nominal Group Technique, our approach aligned with the core elements of iterative expert consultation, structured discussion, and evidence-informed refinement [11]. Particular attention was given to safety considerations, sequencing of interventions, patient selection, and perioperative logistics. Where evidence was lacking, decisions were guided by expert consensus and institutional best practice. The final protocol was piloted in a small cohort of patients, with iterative refinements made in response to feedback and outcomes.

The protocol was submitted for local governance review (as per “New Clinical Procedures or Interventions Policy”) and registered as part of an ongoing quality improvement initiative within the department. Outcomes from the initial cohort were reviewed to inform iterative updates, and protocol compliance was tracked prospectively.

## Protocol

**Fig. 1:**
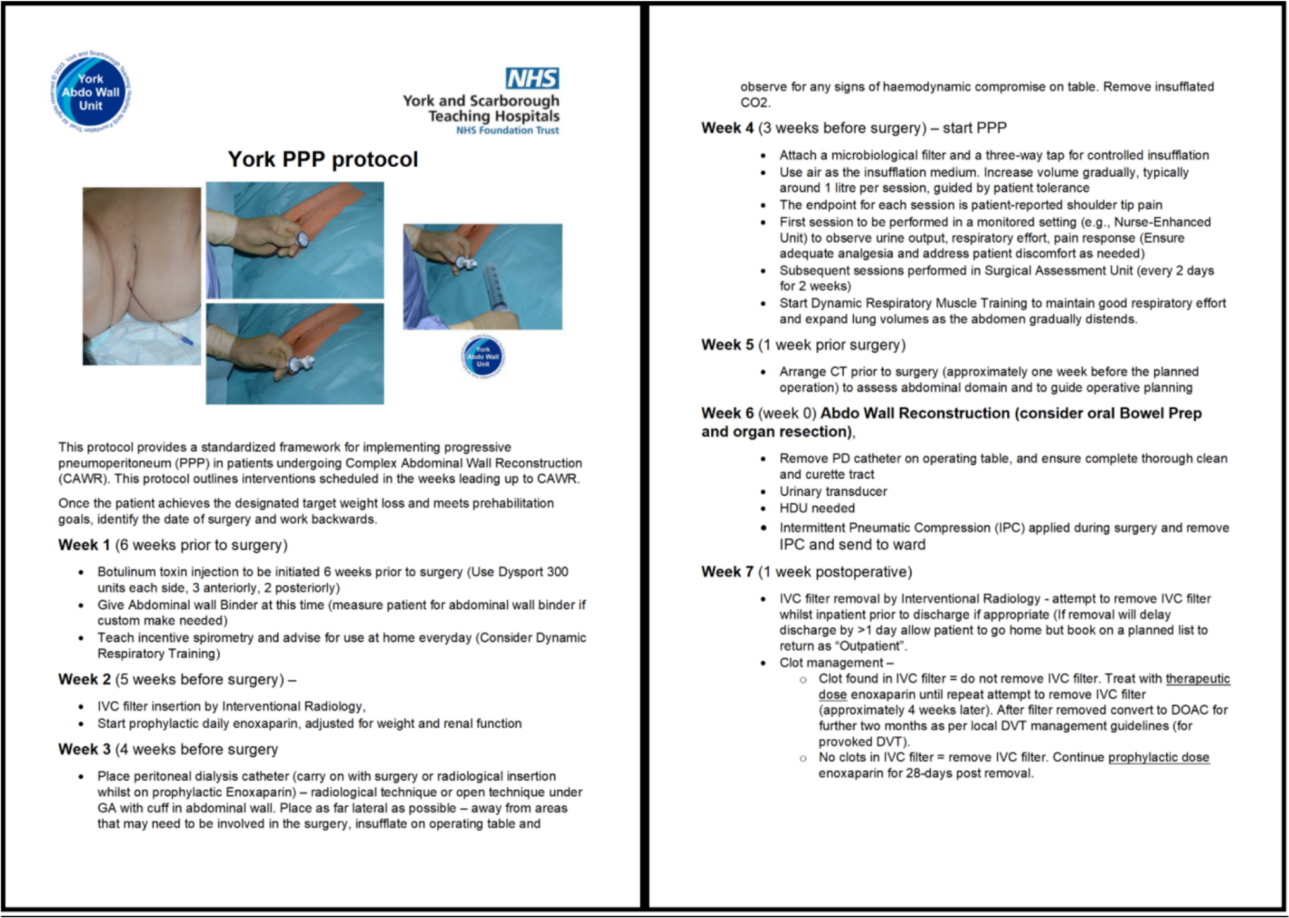
Protocol

This protocol is designed as a sequential, seven-week perioperative pathway for patients undergoing CAWR with PPP as a key component. It includes multidisciplinary interventions to address preoperative conditioning, respiratory training, thromboembolic risk, and surgical planning. The process is initiated once the patient has met baseline optimisation goals, including weight loss targets and prehabilitation benchmarks. Surgery is scheduled, and the protocol is delivered in reverse-engineered fashion from that fixed date, designated “T” (Fig. 1).

### Week 1 (T-6): Neuromuscular conditioning

Six weeks prior to the planned date of surgery, patients receive bilateral intramuscular Botulinum toxin A injections (Dysport, 300 units per side), delivered at five sites per side of the abdomen to promote medialisation of the rectus muscles [7]. This is done in accordance with the Unit’s local Botulinum protocol on a day care unit. At this stage patients are also given an abdominal wall binder if they do not have one, and introduced to incentive spirometry for home use. The binder serves to gently reduce hernia sac contents into the abdominal cavity, thereby encouraging preliminary stretching of the abdominal wall in a controlled manner. Importantly, this stretching effect continues while the binder is in place, and it does not impede the physiological adaptation needed for subsequent PPP. Selected patients are enrolled in Dynamic Respiratory Muscle Training (POWERbreathe®), if they have borderline pulmonary reserve or deconditioning.

### Week 2 (T-5): VTE prophylaxis and IVC filter insertion

Five weeks before surgery, patients are reviewed by Interventional Radiology and undergo insertion of an inferior vena cava (IVC) filter (Cook Celect™ Platinum Vena Cava Filter (manufactured by Cook Medical Europe Ltd., Limerick, Ireland)), due to the risk of venous stasis during progressive abdominal insufflation. Pharmacological VTE prophylaxis is initiated at this point using daily enoxaparin, weight- and renal-adjusted, and is continued throughout the preoperative phase.

### Week 3 (T-4): Peritoneal access

At four weeks preoperatively, a peritoneal dialysis catheter is placed to facilitate serial insufflation. This can be performed either radiologically (via ultrasound ± CT guidance) under local anaesthetic, or surgically under general anaesthetic. The latter is preferred in the case of a patient who has undergone multiple abdominal surgeries involving the right hypochondrium. The catheter is positioned laterally in the right hypochondrium, away from anticipated surgical planes, and anchored to the abdominal wall. Intraoperative insufflation with carbon dioxide is performed at time of insertion to confirm position and haemodynamic tolerance; the gas is subsequently evacuated.

### Week 4—5 (T-3 to T-1): Preoperative Progressive Pneumoperitoneum (PPP)

Progressive pneumoperitoneum is initiated three weeks prior to the scheduled date of surgery:

1. The peritoneal dialysis catheter is checked at each insufflation to ensure the catheter opening is within the abdomen, as surgical emphysema of the abdominal wall has been reported elsewhere [12].
2. Air is insufflated through the peritoneal dialysis catheter. The first insufflation session is conducted in a monitored setting, typically a Nurse Enhanced Unit, to assess haemodynamic response, urine output, respiratory effort, and pain control. Adequate analgesia is administered pre-emptively and titrated as needed.
3. Insufflation is performed using filtered room air, delivered via a three-way tap and microbiological filter (BBraun Sterifix® Injection Filter).
4. The target insufflation volume per session is 1 L, titrated based on patient-reported discomfort, particularly shoulder tip pain, which serves as the endpoint of each session, while ensuring no respiratory compromise. The end of the catheter is covered with a dressing at the end of each session.
5. This is repeated every 48 h over a period of two weeks, for a total of 6–7 sessions, depending on patient tolerance (to onset of shoulder-tip pain). Subsequent insufflations are conducted every two days in a Day unit setting, under trained supervision, with ongoing monitoring of pain and respiratory parameters.

### Week 5 (T-1): Preoperative imaging and final planning

Approximately one week before the planned operation, an abdominal CT scan is obtained to assess the restored abdominal domain, inform fascial closure strategy, and plan the extent of surgical dissection or need for organ resection. The scan is reviewed in a multidisciplinary setting involving the operating surgeon, radiologist, and anaesthetist. At this stage, patients may be advised to commence a bowel preparation regimen depending on intraoperative plans (e.g., anticipated bowel resection).

### Week 6 (T): Surgery

On the day of surgery the peritoneal dialysis catheter is removed intraoperatively, with the catheter tract gently curetted, particularly due to the planned use of mesh and the associated risk of postoperative mesh infection. However, the skin opening is left open to allow for drainage should early signs of infection develop. The patient is then re-prepped and re-draped. Urinary transduction is used intraoperatively to monitor intra-abdominal pressure. Intermittent pneumatic compression (IPC) devices are applied intraoperatively for VTE prophylaxis and subsequently transferred with the patient to the ward postoperatively. Patients are routinely admitted to a High Dependency Unit (HDU) for initial postoperative monitoring.

### Week 7 (T + 1): IVC filter management and VTE follow-up

During the first postoperative week, patients undergo assessment for IVC filter removal by Interventional Radiology. Assessment for IVC filter thrombus is performed by contrast venogram with a catheter in the IVC via a right internal jugular venous puncture. The decision to remove the filter is based on imaging and clinical evaluation: If no thrombus is detected within the filter, it is removed, and prophylactic enoxaparin is continued for 28 days post-removal. If thrombus is identified, the filter is retained, and the patient is commenced on therapeutic-dose enoxaparin and managed in accordance with local protocols for provoked DVT. A follow-up attempt at removal is scheduled approximately four weeks later. Once the filter is successfully retrieved, patients are transitioned to a direct oral anticoagulant (DOAC) for a further two months, in accordance with local protocols for provoked DVT. Where clinically appropriate, filter removal is attempted before discharge. If this would significantly delay discharge (> 1 day), the patient is booked for planned outpatient retrieval.

A detailed summary of the protocol, with weekly interventions and timelines, is presented in Fig. 1 as an appendix to facilitate clinical replication of the protocol.

## Rationale

Each component of the protocol was selected or modified based on emerging evidence, institutional experience and capabilities, and the specific challenges posed by loss of domain, perioperative complications, and anatomical complexity.

### Prehabilitation programme

All eligible patients referred for CAWR are enrolled in our dedicated CAWR prehabilitation programme, delivered in collaboration with a university partner. The programme comprises one-hour group sessions conducted weekly over eight weeks, supervised by a multidisciplinary team including an exercise physiotherapist, an anaesthetist with a specialist interest in exercise physiology and perioperative optimisation, and a senior perioperative nurse with skills in psychological counselling and diet optimisation. When first encountering patients in a clinic they are introduced to written information leaflets on “Getting fitter for CAWR” which covers smoking cessation, obesity management, diet, exercise, and advice on behaviour modification from psychologists [13]. There is structured group physical exercise, aiming to improve physical fitness, functional capacity, and respiratory mechanics, and to achieve realistic and supported weight loss. If patients are identified as smokers or those with poorly controlled diabetes, they are concurrently referred to specialised teams for targeted optimisation of these high risk factors.

### Respiratory prehabilitation

Loss of domain is frequently associated with reduced intra-abdominal volume and diminished diaphragmatic excursion [14]. Rapid reintroduction of herniated viscera into the abdominal cavity during repair may cause abrupt increases in intraabdominal pressure [15], with downstream effects on respiratory mechanics. Preoperative incentive spirometry and dynamic respiratory muscle training aim to improve baseline pulmonary function and prepare patients for the altered ventilatory dynamics following reconstruction [10]. This is especially critical during the progressive pneumoperitoneum phase, where gradual abdominal distension can mimic post-repair physiology [16].

### Use of peritoneal dialysis catheter

**Fig. 2:**
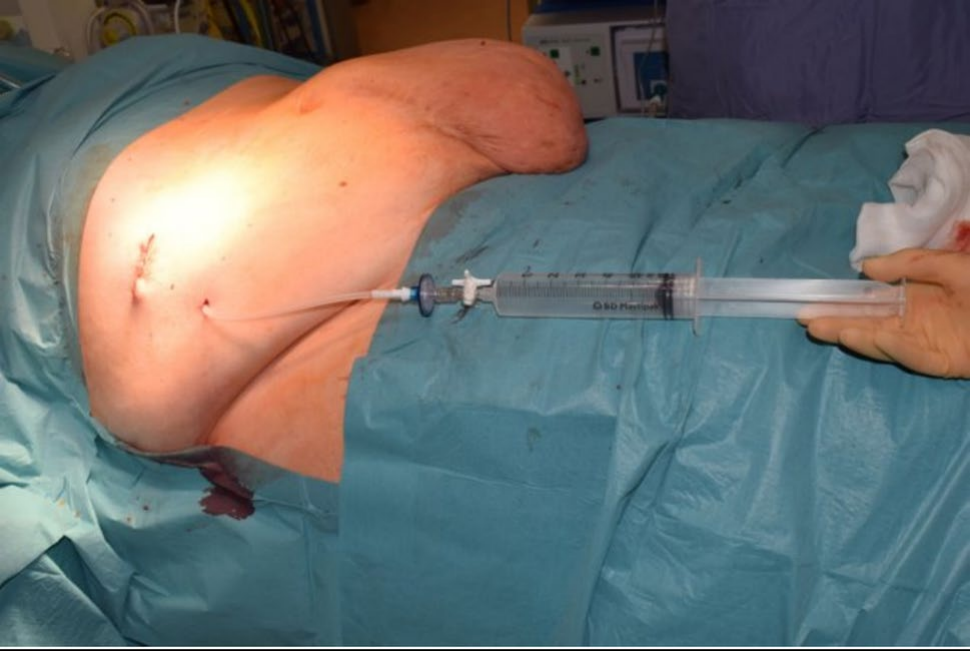
PD catheter insertion, with 3 way tap and microbiological filter shown

The peritoneal dialysis (PD) catheter provides a stable and secure conduit for insufflation. Its established safety profile, low infection risk, and ease of insertion [17] make it a suitable alternative to more invasive access options [18]. Lateral placement ensures it does not interfere with surgical dissection planes or compromise fascial closure. Intraoperative insufflation with air at the time of placement serves to verify tolerance and anatomical positioning, reducing procedural complications during subsequent sessions (Fig. 2).

### Interval imaging for operative planning

Cross-sectional imaging in the week prior to surgery provides valuable information on the extent of abdominal expansion achieved through PPP, the feasibility of fascial closure, and the necessity for adjunctive procedures (e.g. component separation, organ resection). This timing ensures maximal predictive value while allowing for last-minute adjustments to operative strategy [19].

### VTE prophylaxis and IVC filter strategy

**Fig. 3:**
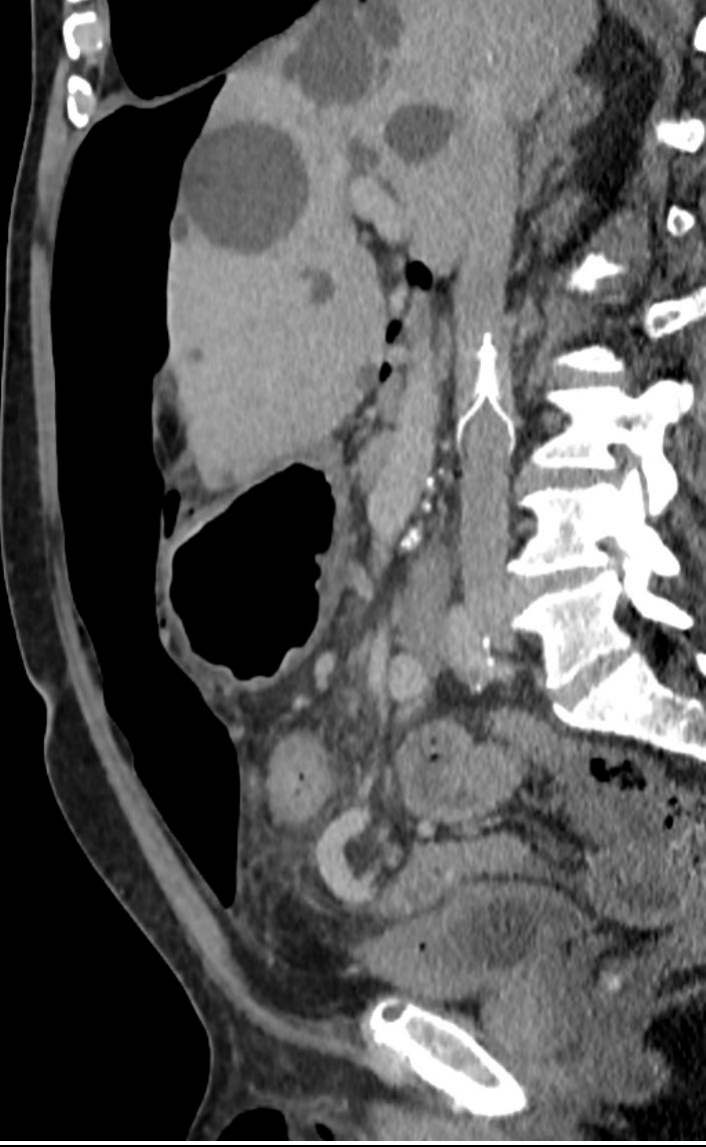
CT reformatted in the sagittal plane showing pneumoperitoneum and IVC filter

The inclusion of both mechanical and pharmacological VTE prophylaxis in our protocol is a deliberate design feature of this protocol, initially based on theoretical considerations outlined in Rosen’s Atlas of Abdominal Wall Reconstruction (1st edition, Chapter 15, page 250), which suggests that increased intraabdominal pressure from progressive insufflation may predispose patients to venous stasis and thromboembolic events [20]. This physiological rationale formed the basis of our early decision to include IVC filter insertion alongside weight- and renal-adjusted prophylactic low molecular weight heparin (LMWH), initiated at week T-5 before PPP commenced (Fig. 3).

**Fig. 4:**
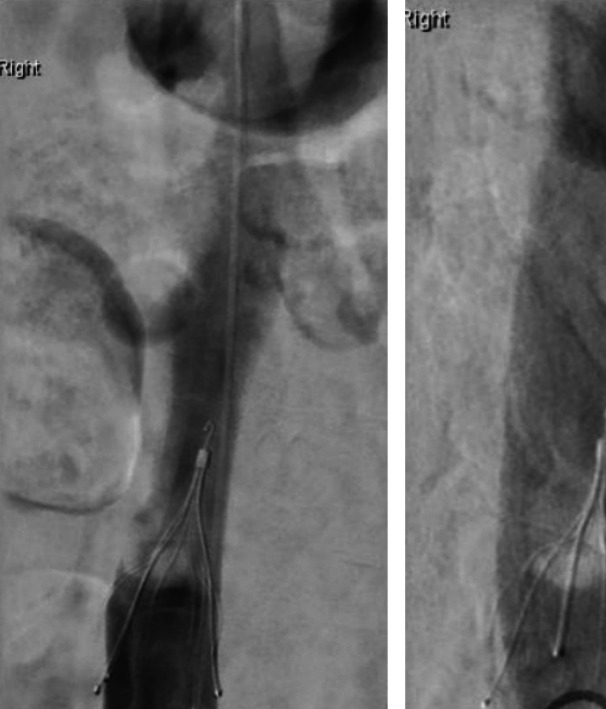
Post-operative cavogram at time of attempted IVC filter removal, demonstrating filling defect which is significant clot

This strategy was further substantiated by our own institutional experience during the initial implementation phase. In a number of cases, thrombus was discovered within the IVC filter at the time of intended postoperative retrieval, with an example shown in Fig. 4. As a result, filters were not removed, and patients were commenced on therapeutic-dose LMWH, followed by a DOAC for a further two months in line with local protocols for provoked DVT [21, 22].

The finalised protocol includes preoperative placement of an IVC filter at week T-5, followed by continuous prophylactic LMWH throughout both the preoperative and postoperative periods. Inpatient assessment for IVC filter retrieval is routinely conducted during the first postoperative week, ideally prior to discharge. In cases where thrombus is identified within the filter, a defined therapeutic escalation pathway is initiated, involving temporary retention of the filter, commencement of therapeutic-dose LMWH, and delayed retrieval after approximately four weeks. If the filter is successfully removed and no thrombus is present, patients continue prophylactic-dose LMWH for 28 days post-removal to mitigate ongoing thromboembolic risk.

To our knowledge, no published PPP protocols incorporate such a comprehensive approach to VTE risk stratification and management. This component of our pathway addresses an important, but often neglected, perioperative risk, ensuring that both mechanical protection and pharmacological prophylaxis are sustained throughout the highest-risk period.

## Discussion

PPP has long been recognised as a valuable adjunct in the surgical management of complex abdominal wall hernias, particularly in patients with significant loss of domain [3–6, 8, 16]. Although its conceptual roots date back almost 100 years [2], formalised and comprehensive protocols remain rare in the literature, with most published accounts focusing on technical aspects in isolation or describing small case series without wider perioperative integration [3].

The protocol described in this manuscript was developed in response to accumulated institutional experience managing patients with large, complex abdominal wall hernias. To date, we have managed over 250 complex abdominal wall hernia patients. We have previously published on “A structured pathway for developing your complex abdominal hernia service: our York pathway” [13]. Over several years, our multidisciplinary team has treated a substantial number of referred cases, many of which presented with recurrent or multiply operated hernias, prior mesh failures, and complex comorbidity profiles. Through this experience we encountered several challenges that shaped the structure of this protocol, including the difficulty of achieving tension-free closure, perioperative respiratory compromise, and thromboembolic events during preoperative preparation. The wide variability in patients’ baseline physiological status, previous surgical history, and hernia morphology required a protocol that incorporated respiratory prehabilitation, radiological planning, and staged insufflation. The surgical approach employed at our institution is highly individualised and includes a protocolised pathway. While formal outcome reporting will follow in a future prospective study, the current protocol reflects an iterative, experience-driven framework that has been shaped by real-world complexity and institutional learning.

Although PPP is considered an effective preoperative adjunct for complex abdominal wall reconstruction, it is not without potential complications. A recent systematic review by Martínez-Hoed et al. (2021) identified an overall complication rate of approximately 12%, predominantly involving minor events such as shoulder-tip pain, abdominal discomfort, and subcutaneous emphysema [5]. However, serious complications are also noted, including visceral injury, pneumothorax, DVT, PE, and respiratory insufficiency. Whilst DVT and PE are infrequently described, their significance is amplified in the context of increased abdominal pressure and potential venous stasis associated with PPP. Respiratory complications, such as restrictive respiratory symptoms, and, in rare cases, respiratory failure, are also documented. Infectious complications, although uncommon, included catheter-related infections and intra-abdominal infections secondary to visceral injury during catheter placement. These reported complications emphasise a need for monitoring and risk mitigation strategies within PPP protocols, and informed our cautious, multidisciplinary institutional approach.

This manuscript presents a structured, multidisciplinary protocol that incorporates PPP into a broader perioperative pathway, integrating surgical, anaesthetic, radiological, and pharmacological strategies. Notably, the inclusion of respiratory prehabilitation, dynamic muscle training, IVC filter planning, and a structured VTE prophylaxis and escalation algorithm sets this protocol apart from existing models. To our knowledge, no other published PPP framework addresses these critical perioperative components in a single cohesive pathway.

The development of this protocol was not purely theoretical. Iterative refinement was informed by direct clinical experience, including the discovery of thrombus within IVC filters at the time of planned removal. These observations prompted the creation of a formalised VTE management pathway, emphasising the importance of combining mechanical protection with pharmacological prophylaxis and escalation where necessary.

Although this protocol was developed at a single institution, it is designed to be adaptable to other settings with access to multidisciplinary care. By codifying practices that are often applied in an ad hoc manner, this pathway offers a practical framework that can be adopted, audited, and studied further [23].

## Limitations

This protocol was developed and implemented at a single tertiary hernia referral centre, and, as such, its generalisability may be limited by variations in institutional resources, multidisciplinary expertise, and access to interventional radiology. The successful application of this pathway relies on coordination across multiple specialties and the availability of high-dependency monitoring, particularly during initial insufflation and the postoperative period. Centres without access to these facilities may require adaptation of the protocol to fit local constraints.

We acknowledge recent literature describing successful implementation of PPP in ambulatory care settings, such as the approach detailed by Donadieu et al. (2025) [24]. Their outpatient protocol described a successful outpatient PPP programme that involves an initial short hospitalisation period for catheter placement and botulinum toxin injection, followed by outpatient sessions occurring three times weekly over approximately three weeks. This ambulatory approach eliminated the routine need for prophylactic anticoagulation or antibiotics, reduced hospital-associated costs, minimised nosocomial infection risks, and allowed patients to maintain daily activities.

However, several key differences justify our more conservative approach. At our institution, patients referred for CAWR typically engage with a structured programme involving obesity management as described earlier. Our use of PPP is structured only when loss of domain is judged as severe. Our preferred way of managing patients is weight loss and fitness interventions, with botox as an adjunct, and PPP only in those situations where it is judged by MDT meeting that closure (even with component separation) would lead to high intraabdominal pressures in the post-operative phase. Additionally, our institutional experience, particularly with cases of thrombus formation identified at IVC filter removal (as demonstrated in Fig. 4), has led to an understanding of the heightened thromboembolic risk associated with the PPP procedure in our specific patient cohort. Insertion of the catheter and index insufflation is performed in hospital, with subsequent insufflations performed in a Surgical Assessment Unit (SAU) in an ambulatory fashion.

We recognise that ambulatory PPP protocols may represent optimal practice for selected patients who are physically active, clinically stable, and can reliably attend outpatient sessions. Nonetheless, our PPP pathway represents a deliberate response to the specific needs and risk profile observed in our patient population, serving as an adaptable framework rather than a universally applicable standard.

At present, this protocol has been piloted in a small cohort of patients as part of routine clinical practice. While early feedback and outcomes have informed iterative refinements, prospective evaluation of its efficacy, safety, and impact on surgical outcomes has not yet been completed. Data on key endpoints—such as fascial closure rates, complication profiles, hospital stay, and patient-reported quality of life—are being collected and will be presented in a subsequent study. Therefore, this manuscript should be regarded as a protocol description rather than an outcomes study.

Further, while several protocol components (e.g., botulinum toxin injection, PPP, IVC filter insertion) are supported by published literature, the exact combination and timing of interventions used in this protocol have not been validated in a controlled trial. As such, the protocol represents a synthesis of best available evidence and expert consensus, rather than an evidence-based guideline.

Finally, the protocol does not yet incorporate formalised patient-reported outcome measures (PROMs), although this remains a priority for future iterations. Understanding the patient experience (particularly regarding tolerability of insufflation, respiratory training, and psychological preparedness) will be essential to assessing the holistic value of the PPP pathway.

## Conclusion

PPP remains an underutilised but valuable technique in the management of patients undergoing CAWR, particularly those with significant loss of domain. Despite its longstanding conceptual basis, few published protocols offer a comprehensive, structured approach that integrates PPP into the broader perioperative care pathway.

We provide a multidisciplinary protocol that is reproducible and encompasses prehabilitation, thromboembolic prophylaxis, peritoneal access, insufflation strategy, imaging, operative planning, and postoperative management. By systematically addressing the mechanical, physiological, and logistical challenges associated with CAWR, this protocol aims to improve the safety and efficacy of reconstruction in anatomically and clinically complex patients.

This protocol is not a hagiarchy but may serve as a foundation for standardisation across other high volume units, and we encourage further evaluation through prospective multicentre implementation. Incorporation of patient reported outcomes and longer term follow-up will be key to assessing the full impact of this structured pathway on surgical success, recovery experience, and quality of life.

## Ethics

This protocol was developed and implemented as part of a local quality improvement initiative within a tertiary surgical centre. As such, it did not require formal ethical approval under UK Health Research Authority (HRA) guidelines, as no experimental interventions were introduced beyond standard clinical practice. All patients undergoing treatment within the protocol pathway provided informed consent for each component of care, including the use of botulinum toxin, peritoneal catheter placement, and IVC filter insertion.

In addition, where clinical photography was obtained to illustrate the protocol, written patient consent was obtained for the use of these images in both academic and research dissemination, including publication. Patient identities were anonymised, and no identifying information is included in the manuscript or associated materials.

## Data Availability

Not applicable.
